# Formation of the *Francisella tularensis* Biofilm is Affected by Cell Surface Glycosylation, Growth Medium, and a Glucan Exopolysaccharide

**DOI:** 10.1038/s41598-019-48697-x

**Published:** 2019-08-22

**Authors:** Anna E. Champion, Kelly C. Freudenberger Catanzaro, Aloka B. Bandara, Thomas J. Inzana

**Affiliations:** 1 Center for Molecular Medicine and Infectious Diseases, Department of Biomedical Sciences and Pathobiology, Virginia-Maryland College of Veterinary Medicine, Virginia Tech, Blacksburg, VA 24061 USA; 2 Virginia Tech Carilion School of Medicine, Roanoke, VA 24016 USA; 3 Present Address: Long Island University, College of Veterinary Medicine, 216 Roth Hall, Brookville, NY 11548 USA

**Keywords:** Bacteriology, Biofilms, Pathogens

## Abstract

Biofilms are matrix-associated communities that enable bacteria to colonise environments unsuitable for free-living bacteria. The facultative intracellular pathogen *Francisella tularensis* can persist in water, amoebae, and arthropods, as well as within mammalian macrophages. *F*. *tularensis* Types A and B form poor biofilms, but *F*. *tularensis* mutants lacking lipopolysaccharide O-antigen, O-antigen capsule, and capsule-like complex formed up to 15-fold more biofilm than fully glycosylated cells. The Type B live vaccine strain was also 50% less capable of initiating surface attachment than mutants deficient in O-antigen and capsule-like complex. However, the growth medium of all strains tested also influenced the formation of biofilm, which contained a novel exopolysaccharide consisting of an amylose-like glucan. In addition, the surface polysaccharide composition of the bacterium affected the protein:DNA:polysaccharide composition of the biofilm matrix. In contrast, *F*. *novicida* attached to surfaces more efficiently and made a more robust biofilm than Type A or B strains, but loss of O-antigen or capsule-like complex did not significantly affect *F*. *novicida* biofilm formation. These results indicated that suppression of surface polysaccharides may promote biofilm formation by *F*. *tularensis* Types A and B. Whether biofilm formation enhances survival of *F*. *tularensis* in aquatic or other environmental niches has yet to be determined.

## Introduction

*Francisella tularensis* is a gram-negative, facultative intracellular bacterium that can infect numerous mammals, arthropods and other eukaryotes, and is the etiologic agent of tularemia or “rabbit fever”^1–3^. *F*. *tularensis* is categorised by the Centers for Disease Control and Prevention (CDC) as a Tier I select agent due to its capacity to cause fatal disease by as few as 10 cells and has the capability to be weaponised. Isolates that are most commonly responsible for disease belong to subsp. *tularensis* (Type A) and subsp. *holarctica* (Type B). Each year in the U.S. there are over 100 cases of naturally occurring tularemia, predominately by Type A strains, with the majority of transmissions occurring through tick bites^4^. *F*. *tularensis* subsp. *holarctica* is less virulent to humans than subsp. *tularensis*, and is more common in Europe where it is not uncommon for over one thousand human cases to occur in a given year^5^. The route of infection for the majority of cases in northern Europe is suspected to be mosquito-borne, as most patients are near water and report mosquito bites^6^. Most mammals are susceptible to infection, and typically die from the disease, although some animals are more resistant to disease and can clear the bacteria^6^; therefore, it is likely that *F*. *tularensis* can persist in nature in non-mammalian sites. Studies using molecular typing methods have shown that *F*. *tularensis* can persist in water, mud, and insects^7–9^, supporting persistence of *F*. *tularensis* in the environment, but also raising question as to how this fastidious bacterium does persist in such sites. Most of the current research on *F*. *tularensis* has focused on mammalian infection, growth in macrophages and other cells, host immune response, and vaccine development, but other facets of the biology of this bacterium require investigation to more thoroughly understand the life cycle and transmission of *F*. *tularensis*.

In order to persist in terrestrial and aquatic habitats, especially where nutrients are limited, bacteria commonly form a biofilm. A biofilm is an aggregated microbial community that forms within an extracellular polymeric substance, normally composed of exopolysaccharide (EPS), extracellular DNA (eDNA), and proteins^10^. *F*. *novicida* is a distinct species or subsp. of *F*. *tularensis* that is of low virulence for humans, but highly pathogenic for mice, and antigenically distinct from subsp. *tularensis* and *holarctica*. *F*. *novicida* readily forms biofilms on a variety of surfaces including plastics, glass, and crab shells^11^. In contrast, *F*. *tularensis* subsp. *tularensis* and subsp. *holarctica* generally do not form a substantial biofilm, although they are capable of developing some biofilm on polystyrene 96-well plates^12^.

Despite their genetic similarity, the less virulent *F*. *novicida* strains differ from Types A and B (A/B) strains in their polysaccharide surface components. The composition and antigenic reactivity of the lipopolysaccharide (LPS) O-antigen of Types A/B strains is distinct from that of *F*. *novicida*^13^. Type A strain SchuS4 and Type B live vaccine strain (LVS) have O-Ag structures that consist of a 4-glycose unit of two internal carbohydrate residues (α-D-GalNAcAN–α-D-GalNAcAN) and two peripheral residues (β-D-Qui4NFm and β-D-QuiNAc)^14^, while *F*. *novicida* O-Ag shares the same two internal residues, but has different terminal residues (α-D-GalNAcAN and α-D-QuiNAc4NAc)^13^. Genomic analysis of the O-Ag gene clusters (*wbt* locus) of the two species confirms that while genes encoding for proteins responsible for O-Ag synthesis in Types A/B strains are identical, the *F*. *novicida* O-Ag locus has fewer and some different genes, and a lower G + C ratio^15,16^. The LPS is an important virulence factor for all subspecies, as it protects the bacterium against innate host defenses, such as complement-mediated killing^17^, and mutants lacking O-Ag are highly attenuated in mice^15,18.^ Subspecies *tularensis* and *holarctica* also produce an O-Ag capsule, but if only the O-Ag capsule is absent, the bacteria remain serum-resistant^19^. Types A/B strains and *F*. *novicida* also produce a novel capsule-like complex (CLC) containing glycosylated proteins^20,21^. Whether the differences in surface glycosylation between Types A/B strains and *F*. *novicida* contribute to their differences in capability to form biofilm or development of the biofilm matrix has yet to be determined.

In this investigation the capability of the more virulent Types A/B strains to form a biofilm in comparison to glycose-deficient mutants and *F*. *novicida* was examined. Whereas wildtype *F*. *novicida* made a robust biofilm within 2–3 days of incubation *in vitro*, Types A/B strains required at least 10 days to form a similar biofilm. However, loss of the LPS O-Ag and/or loss of the capability to glycosylate CLC proteins resulted in enhanced biofilm formation sooner by Types A/B strains, but not *F*. *novicida*. Furthermore, following *F*. *tularensis* biofilm formation the EPS from the matrix material was isolated and identified as glucan, making this the first report of glucan production by *F*. *tularensis*. We hypothesise that *F*. *tularensis* can phase vary its surface polysaccharides to successfully transition between a phenotype that forms a biofilm, which may enhance survival in the environment, and a planktonic phenotype that is a facultative intracellular pathogen in the mammalian host.

## Results

### Effect of incubation time and bacterial surface components on attachment and biofilm development

**Figure 1:**
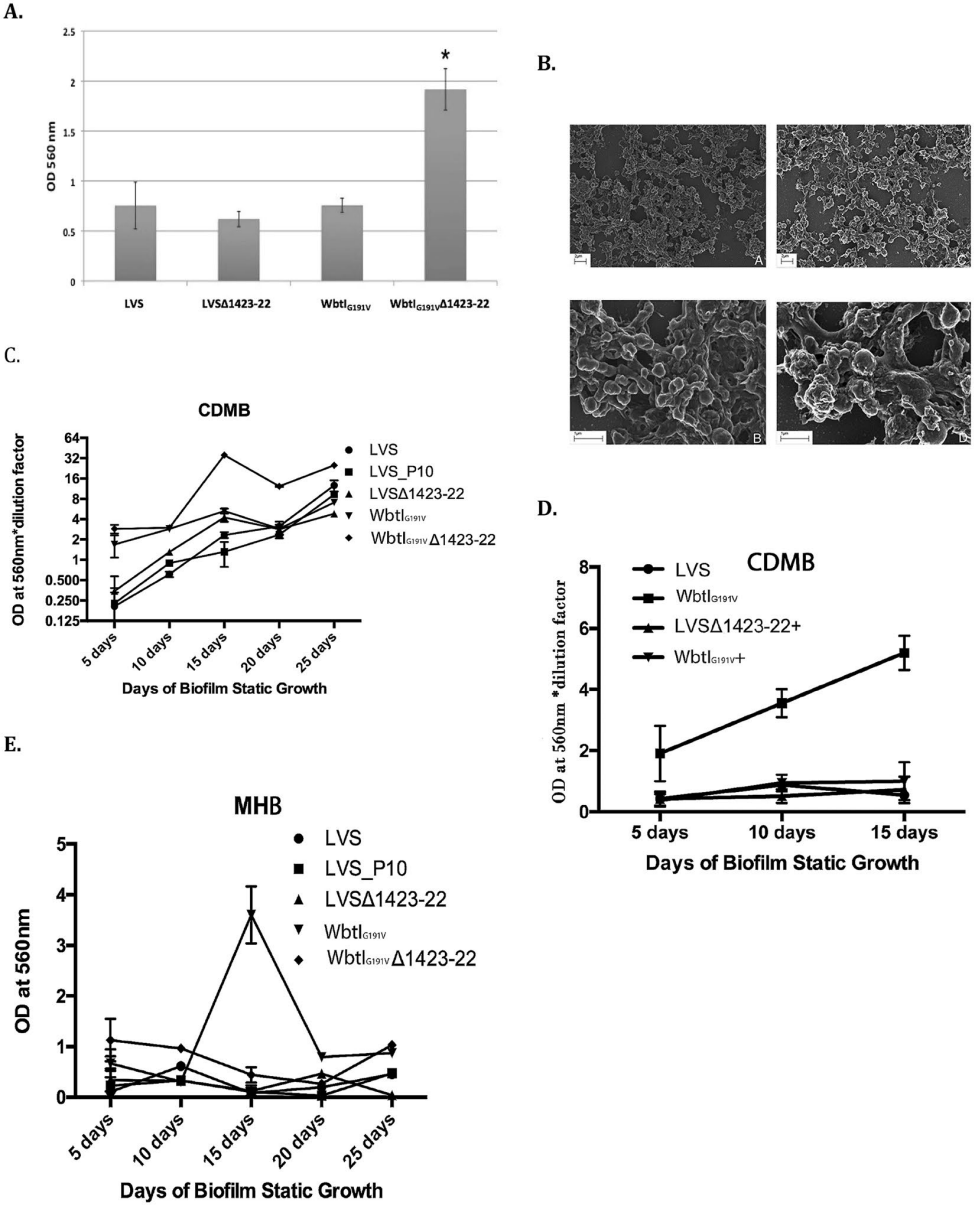
Biofilm development by LVS and surface antigen mutants: attachment and static growth. (**A**) CV attachment assay. Bacteria were grown to stationary phase, inoculated into 96-well plates in triplicate, incubated for 2 hours at 37 °C, washed, and stained with CV to detect cells that have attached. The absorbance was determined at 560 nm after the CV was solubilised with 95% ethanol. *p value < 0.005. (**B**) SEM of *F*. *tularensis* biofilms. Biofilms were grown on silicone disks for 5 days and carbon-coated (panels and strains: (A,B), LVS; (C,D), LVSΔ1423-22). After 5 days growth, no difference was seen in overall attachment or biofilm formation. At increased magnification the surface of LVSΔ1423-22 appeared more granular than the parent, which could be the result of CLC deficiency. (**C**,**D**,**E**) Effect of growth medium on biofilm formation by LVS, glycose-deficient mutants, and complemented strains. C, biofilm formation by parent and mutant strains grown in CDMB; D, biofilm formation by parent and complemented mutant strains (LVSΔ1423-22+ and WbtI_G191V_+) grown in CDMB with O-Ag mutant WbtI_G191V_ included for comparison; E, biofilm formation by parent and mutant strains grown in MHB. A marked increase in biofilm formation by WbtI_G191V_Δ1423-22 occurred in MHB medium after 15 days incubation that was not observed following growth in CDMB.

Static attachment of LVS to a polyvinyl surface occurred within 1 hour (Fig. 1A), and micro-colony and some multi-layering of cells occurred after 5 days incubation (Fig. 1B). However, there was no significant difference between the number of LVS cells and LVS mutants (Table 1) deficient in O-antigen (Ag) (WbtI_G191V_) or CLC (LVSΔ1423-22) that attached to polyvinyl 96-well plates (Fig. 1A), or the time required for attachment (1, 2, or 4 hours; data not shown). However, the LVS double mutant lacking O-Ag and CLC (WbtI_G191V_Δ1423-22) attached significantly better (*p* = 0.0101) (about 2-fold) than the parent LVS strain or mutants deficient in only O-Ag (*p* = 0.0025) or only CLC (*p* = 0.0019) (Fig. 1A).

**Table 1:** Strains used in this study.

Strain	Characteristics	LPS/O-Ag	O-Ag capsule	CLC	Attenuated	Source/Reference
*F*. *tularensis* LVS (Type B)	Attenuated Live Vaccine Strain	+	+	+	N	Dr. May Chu, CDC
LVS_P10	LVS subcultured 10 consecutive days in broth CDMB	+	+	+++	N	^20^
LVSΔ1423-22	LVS deficient in CLC due to deletion of genes encoding for glycosyltransferases	+	+	−	Y	^20^
WbtI_G191V_	O-Ag mutant of LVS due to point mutation in *wbtI*	−	−	+	Y	^18^
WbtI_G191V__P10	WbtI_G101V_ subcultured 10 consecutive days in CDMB	−	−	+++	Y	^20^
WbtI_G191V_Δ1423-22	O-Ag and CLC deficient double mutant. WbtI_G191V_ with deletion of FTL_1423 and 1422	−	−	−	Y	^20^
WbtI_G191V_^+^	Complemented strain of respective mutant with O-Ag restored	+	+	+	ND^b^	^18^
LVSΔ1423-22^+^	Complemented strain of respective mutant with CLC restored	+	+	+	ND	^20^
KY99-3387	Wild Type B strain	+	+	+	N	BEI Resources
KY99-3387_P10	KY99-3387 subcultured 10 consecutive days in broth CDMB	+	+	+++	N	This work
*F*. *novicida* U112	Parent *F*. *novicida* strain	+	ND	+/−	N	Dr. Karen Elkins, FDA
*F*. *novicida*_P10	Strain U112 subcultured 10 consecutive days in CDMB	+	ND	+++	N	^21^
*F*. *novicida*Δ1212-18	CLC-deficient strain of U112 due to loss of FTN_1212-1218 in CLC glycosylation locus	+	ND	−	Y	^21^
*F*. *tularensis* SchuS4 (Type A)	Virulent Type A strain parent	+	+	+	N	Dr. Mark Wolcott, USAMRIID
*F*. *tularensis* TI0902 (Type A)	Highly virulent Type A strain parent	+	+	+	N	^62^
*F*. *tularensis* TIGB03	Chemical mutant of TI0902 lacking O-Ag	−	−	+	Y	^25^

(+, present; +++, enhanced; −, not present).

^a^Not determined.

**Figure 2:**
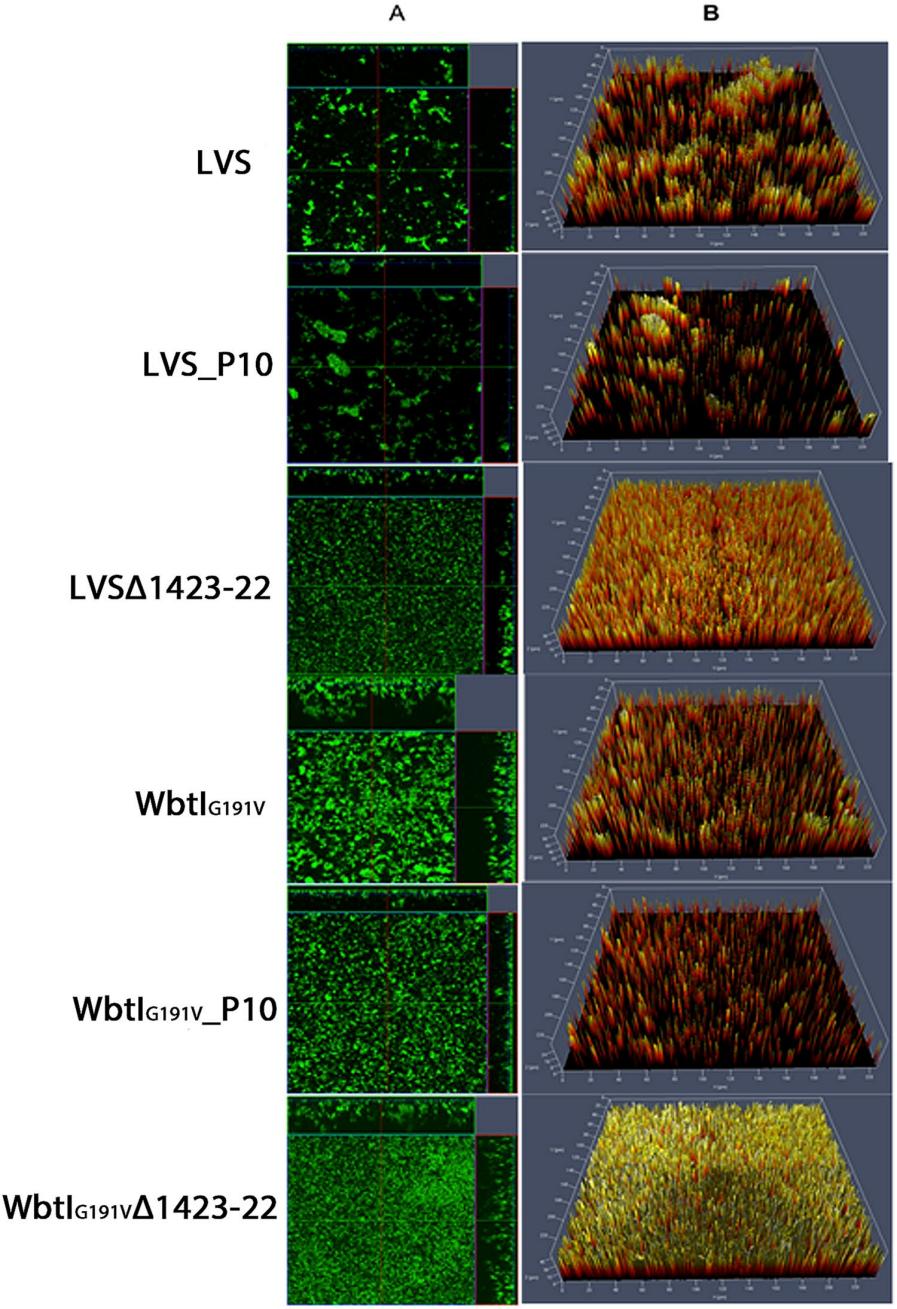
Comparison of biofilm formation by *F*. *tularensis* LVS, WbtI_G191V_ (LPS O-Ag mutant), LVSΔ1423-22 (CLC mutant), and O-Ag and CLC double mutant before and after passage in CDMB by confocal laser scanning microscopy. Panel (A) contains orthogonal sections showing horizontal (z) and side views (x and y) of reconstructed three-dimensional biofilm images at a magnification of 25x. Biofilms were stained with Syto9 showing both live and dead bacterial cells. Panel (B) shows topographical images of biofilm growth of the parent LVS and its surface structure mutant strains. Loss of surface carbohydrate was associated with denser biofilms, but subculture passage in CDMB resulted in thinner biofilms.

Previous studies of biofilm formation by *F*. *novicida* and *F*. *tularensis* strains measured biofilm formation after seven days or less^11,22,23^. However, we observed that at least 10–15 days incubation was required for optimum biofilm development by LVS, and to fully differentiate biofilm development between LVS and LVS mutants deficient in O-Ag or glycosylation of CLC surface structures (WbtI_G191V_, LVSΔ1423-22, and WbtI_G191V_Δ1423-22, respectively). However, production of a clear biofilm by the O-Ag mutants was evident within 5 days of incubation. Nonetheless, a mature biofilm by all strains tested required about 15 days incubation, at which time differences in thickness and overall mass between the parent and each mutant were maximised (Fig. 1C). Both the single mutant (LVSΔ1423-22) and double mutant (WbtI_G191V_Δ1423-22) with deletions in the polysaccharide locus that glycosylates CLC (CLC-deficient) formed 2 to 4-fold and 15-fold more biofilm, respectively, than their parent strain by day 15 (Fig. 1C). After 15 days incubation there was a reduction in the more mature biofilms, indicating that dispersal of planktonic cells occurred; after about 20 days the biofilm cycle began again with reattachment of cells and biofilm formation (Fig. 1C). Complementation of the O-Ag mutation or the CLC mutation *in trans* completely reversed the enhancement in biofilm formation (Fig. 1D). Furthermore, LVS formed 46% more biofilm than the CLC-enhanced strain LVS_P10 (LVS subcultured in Chamberlains Defined Medium Broth (CDMB) for 10 consecutive days) after 15 days incubation (Table 2). Therefore, cell attachment and the amount of biofilm formed was inversely proportional to the amount of cell-associated carbohydrate on the bacterial surface. These differences in biofilm formation by parent, mutants, and CLC-enhanced strains were also evident by confocal laser scanning microscopy (CLSM) (Fig. 2), particularly after 15 days of incubation. LVS, and to a greater degree LVS_P10, developed more sparse micro-colonies, whereas the mutants, particularly WbtI_G191V_Δ1423-22, developed more confluent, thicker biofilms. However, O-Ag-deficient variant WbtI_G191V__P10, which is enhanced for CLC, also produces a less dense biofilm compared to the non-subcultured mutant (Fig. 2). The mutants that made a more robust biofilm did not increase the amount of substrata surface coverage as much as they increased the formation of dense, cellular aggregates that adhered to the surface, as well as floating pellicles.

**Table 2:** Fold-difference in biofilm development^a^ in CDM or MH compared to LVS.

	Day 10		Day 15	
	CDMB	MHB	CDMB	MHB
LVS	1	1	1	1
LVS_P10	1.5 ± 0.20	−2.5 ± 0.01*	−1.8 ± 0.4^*^	1.3 ± 0.75
LVSΔ1423-22	2.1 ± 0.16	−2.5 ± 0.01*	1.8 ± 0.1	1.56 ± 0.91
LVSΔ1423-22+^b^	−1.05 ± 0.17	ND^c^	−1.48 ± 0.44*	ND
WbtI_G191V_	4.6 ± 0.17**	−2.7 ± 0.01	2.3 ± 0.1	45.2 ± 0.138**
WbtI_G191V_+	−1.38 ± 0.20	ND	−1.48 ± 0.16	ND
WbtI_G191V__Δ1423-22	4.9 ± 0.10**	1.23 ± 0.1	15.3 ± 0.5**	5.5 ± 0.34**

^a^Fold-difference calculated from OD560nm of CV assays.

^b^+-strain with mutation complemented.

^c^ND-Not determined.

^*^Indicates P < 0.05; **Indicates P < 0.01.

**Figure 3:**
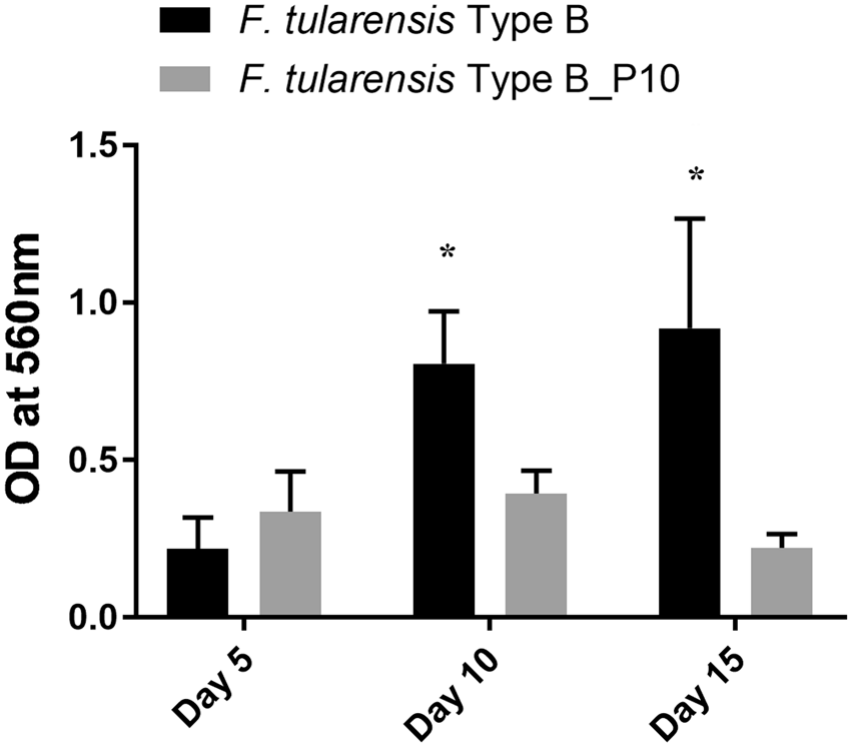
Biofilm formation by a *F*. *tularensis* subspecies *holarctica* wildtype strain following subculture passage in CDMB. A wildtype Type B strain was incubated in microtiter plates under biofilm forming conditions before and after 10 daily subculture passages in CDMB. After 5, 10, or 15 days incubation the wells were rinsed to remove planktonic cells, stained with CV, and the CV solubilized with ethanol and the absorbance determined at 560 nm. Five to six replicates of the parent and the passed variant were tested at each time period. *Indicates a statistically significant difference (*P* < 0.05).

LVS is not a wildtype Type B strain, as it has been extensively subcultured and is known to contain multiple mutations within its genome^24^. Therefore, wildtype Type B strain KY99-3387 was subcultured in CDMB for 10 passages to obtain KY99-3387_P10 to enhance CLC expression (attempts to isolate a grey, O-antigen-deficient variant of strain KY99-3387 were unsuccessful). As for LVS, strain KY99-3387_P10 formed significantly less biofilm by 10 and 15 days incubation than non-subcultured strain KY99-3387 (Fig. 3), supporting the evidence that growth conditions that enhance surface carbohydrate expression reduce biofilm formation of type B strains.

### Effects of growth medium on biofilm development by *F*. *tularensis*

Growth curves of all *F*. *tularensis* strains and mutants grown in either CDMB or Mueller-Hinton broth (MHB) were similar (data not shown). However, the amount of biofilm formed (as determined by crystal violet (CV) assay) was greatly enhanced when the bacteria were grown in CDMB (Fig. 1C) compared to MHB (Fig. 1E) for all strains by post-inoculation days 10 and 15 (Table 2) and beyond. The fold-difference in biofilm formed increased the most for the mutant lacking both O-Ag and CLC (WbtI_G191V_Δ1423-22) in CDMB, particularly 15 days post-inoculation when compared to the parent (15.3-fold in CDMB). Overall there was little biofilm formed by the parent, CLC-enhanced parent, or mutants over 25 days of growth in MHB. The exception was mutant WbtI_G191V_Δ1423-22, which spiked a 5.5-fold increase in biofilm formation by 15 days incubation in MHB, but was reduced again by 20 days incubation (Fig. 1E). Any increase in biofilm formation by the mutants was eliminated when the mutation was complemented (Table 2). These differences indicated that both the growth medium, and the presence or absence of bacterial surface polysaccharides (O-Ag and CLC), influenced biofilm development in *F*. *tularensis*.

### Extraction of the CLC from biofilm cells

**Figure 4:**
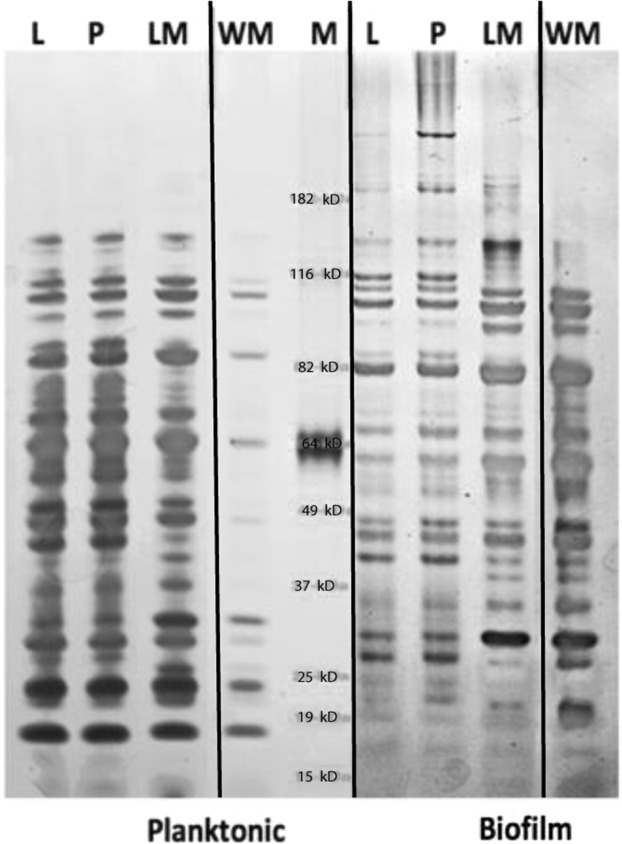
Electrophoretic protein profiles of LVS variants or mutants grown as a biofilm or are planktonic cells from the biofilm broth supernatant. Bacterial strains were grown as a biofilm in CDMB for 25 days, the medium above the biofilm was removed, and the planktonic cells harvested by centrifugation. Planktonic cells in the medium and cells in the biofilm were extracted with 1 M urea. The protein profiles were resolved by SDS-PAGE on 4-12% gels and stained with silver. Strains: L, LVS; P, LVS_P10; LM, LVSΔ1423-22; WM, WbtI_G191V_Δ1423-22; M, Molecular size standards. The figure is a compilation of four separate gels containing samples prepared during the same experiment (left two are from planktonic phase cells and right two are from biofilm cells), which have been cropped to present the most relevant information (original shown in Supplementary Fig. S3). Precipitates of the broth medium are not shown. Lanes of WbtI_G191V_ (W) have been deleted from the planktonic cells (far left) and from the biofilm cells (far right) due to lack of differences with WbtI_G191V_Δ1423-22 (WM). Repeated subculture in CDMB had little effect on protein composition, but loss of surface glycoses, particularly O-Ag, or growth as a biofilm substantially altered the protein profile.

LVS and LVS_P10 cells grown as biofilms both produced high molecular size (HMS) components that have been associated with the CLC^20^ (Fig. 4, Biofilm plate; L [LVS] and P [LVS_P10], respectively). These HMS components were clearly more prominent following repeated subculture of LVS in CDMB, which greatly enhances CLC formation^20^. However, the HMS bands were diminished in extracts from the biofilm of LVSΔ1423-22 (LM) and absent from the biofilm of WbtI_G191V_Δ1423-22 (WM), from which biofilms were more prominent, but CLC is not produced^20^. HMS bands were also not present in any of the bacteria or mutants in planktonic phase, from which CLC is not formed^20^. There were also clear differences in the overall protein profiles of planktonic bacteria and biofilm bacteria, as well as in O-Ag and CLC mutants (Fig. 4). Therefore, growth as a biofilm and loss of surface carbohydrates also affected the protein profile of *F*. *tularensis*.

### Composition and analysis of the extracellular matrix exopolysaccharide (EPS)

**Figure 5:**
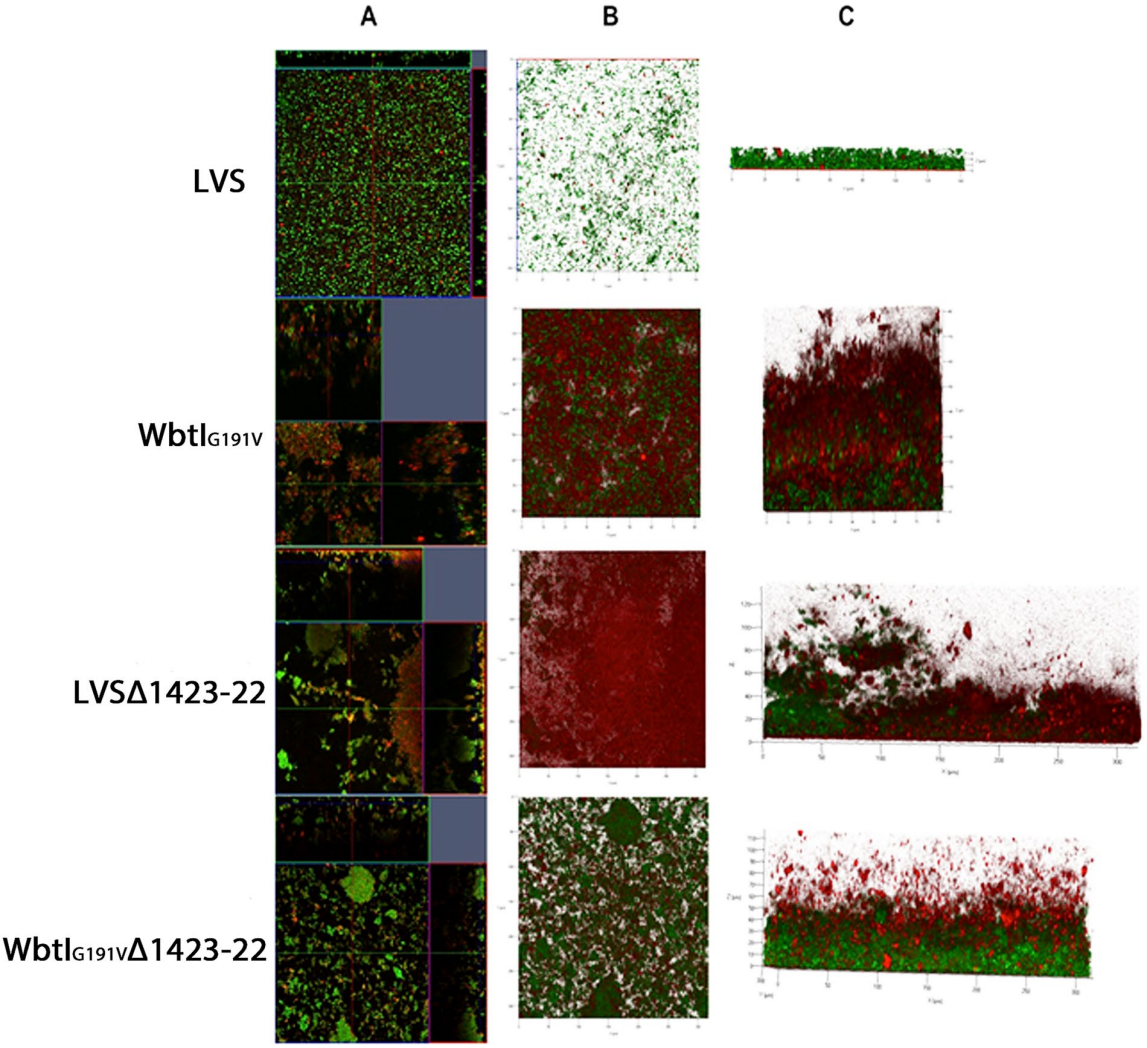
Comparison of biofilm matrix development by *F*. *tularensis* LVS and its surface structure mutants by CLSM. Panel (A) shows orthogonal sections of horizontal (z) and side views (x and y) of reconstructed three-dimensional biofilm images at a magnification of 25x. Nucleic acids were stained with Syto9 (green) and the matrix EPS stained with Texas Red-ConA (red). Panel (B) is an overview (x and y) view of the reconstructed three-dimensional z-stack of each biofilm growth on the coverslip. Panel C is the side view of the three-dimensional z-stack. Each picture was obtained from biofilms grown for 15 days.

EPS was extracted from WbtI_G191V_Δ1423-22, which made the most biofilm. The extracted EPS was visualised following sodium dodecyl sulfate-polyacrylamide gel electrophoresis (SDS-PAGE) and staining with Pro-Q Emerald 300 fluorescent carbohydrate stain. The EPS appeared as a diffuse smear, but included large molecular size material >200-kDa that was not visible with silver stain alone (data not shown). Based on gas chromatography-mass spectrometry (GC-MS), the composition of the purified EPS was determined to be 99.9 mole% glucose and 0.1 mole% mannose. Subsequent linkage analysis indicated that 54% of the glucose residues were terminal and that 33% were 1,4-linked and that 1.6% of the glucose was 1,6-linked, indicating the composition of the EPS was consistent with amylose (Supplementary Fig. S1). An additional 4.4% of the EPS contained 1,4-linked mannose residues. The presence of 1,6-linked poly-*N*-acetylglucosamine (PNAG) was not identified in the biofilm based on GC-MS, lack of reactivity by immunoblotting with antibodies to PNAG (Sigma-Aldrich, St. Louis, MO), or by fluorescence microscopy with wheat germ agglutinin (lectin to PNAG) (data not shown). Texas Red-conjugated Concanavalin A (ConA), which binds to α-D-mannosyl and α-D-glucosyl groups, reacted strongly with LVS, WbtI_G191V_, and WbtI_G191V_Δ1423-22 biofilms (Fig. 5C). An increase in the amount of EPS/matrix (red) was evident in the biofilms of both mutants. However, the biofilm matrix could not be clearly resolved by CLSM in conjunction with ConA due to the thickness of the matrix. A BLASTP search of proteins responsible for glycogen expression and regulation in *Escherichia coli* (GlgXBACP) identified genes with substantial identity in *F*. *tularensis* SchuS4 (Table 3). The most closely related proteins were from *Clostridium* spp., but also from *Massilia* spp., *Lachnospiraceae* spp., *Burkholderia* spp., and *Lachnospiraceae* spp. Of interest was that *glgC* is described as a pseudo gene in Type A *F*. *tularensis*, but is a functional glucose-1-phosphate adenylyltransferase in Type B LVS.

**Table 3:** *Francisella tularensis* Type A strain SchuS4 genes with amino acid identity to glycogen synthesis proteins in heterologous bacteria.

*F*. *tularensis*	Gene symbol	Putative gene product	Percent amino acid	Gene Size (bp)
Locus tag^a^			identity to:^b^	
FTT_0412	*pulB*	type I pullulanase	*Clostridium* spp.-50% 3212	3212
FTL_RS02530			*F*. *tularensis* LVS-97%	
FTT_0413	*glgB*	1,4-α-glucanotransferase	*Clostridium perfringens*-63%	1,922
FTL_RS02535		glycogen branching enzyme	*F*. *tularensis* LVS-99%	
FTT_0414	*pgm*	α-D-glucose phosphoglucomutase	*Massilia* spp.-68%	1,634
FTL_RS02540			*F*. *tularensis* LVS-99%	
FTT_0415	*glgC*	Pseudo		1,269
FTLRS02545		glucose-1-phosphate adenylyltransferase	*Lachnospiraceae* spp.-63%	1,271
			*F*. *tularensis LVS*-99%	
FTT_0416	*glgA*	glycogen synthase	*Burkholderia ubonensis*-63%	1,469
FTL_RS02550			*F*. *tularensis* LVS-96%	
FTT_0417	*malP*	maltodextrin phosphorylase	*Lachnospiraceae bacterium*-63%	2,273
FTL_RS02555			*F*. *tularensis* LVS-99%	
FTT_0418	*malA*	4-α-glucanotransferase (amylomaltase)	*Clostridium* spp.*-*53%	1,463
FTL_RS02560			*F*. *tularensis*LVS-97%	

^a^Type A SCHUS4 locus tag, followed by the Type B LVS locus tag listed underneath.

^b^Genus other than *Francisella* with the closest amino acid identity.

**Figure 6:**
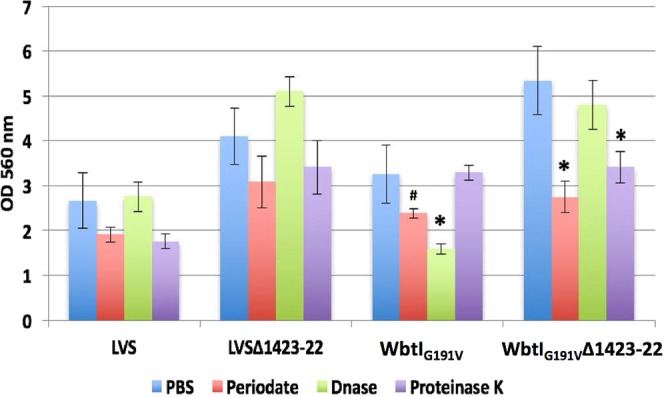
Effect of periodate, DNAse, or Proteinase K on biofilm integrity. Ten-day old biofilms were treated with sodium *m*-periodate, DNase, Proteinase K, or PBS for two hours at 37 °C, washed with distilled water and left to dry overnight before CV staining. Only WbtI_G191V_ and WbtI_G191V_Δ1423-22 contained significantly less biofilm (^#^*p* = 0.0246, *p = 0.0055) after periodate treatment when compared to untreated biofilm from the same strain, indicating these strains may produce more of the glucan EPS in their biofilms compared with the LVS parent strain. The WbtI_G191V_ biofilm only was significantly diminished after treatment with DNase (*p = 0.0190), whereas only the WbtI_G191V_Δ1423-22 biofilm was significantly affected by treatment with Proteinase K (*p = 0.0162).

Enzymatic treatment of the biofilms using sodium *m*-periodate reduced biofilm mass for all strains, but there was a significant reduction in biofilm for only strains WbtI_G191V_ (*p* = 0.0246) and WbtI_G191V_Δ1423-22 (*p* = 0.0055) compared to treatment of the biofilms with phosphate buffered saline, pH 7.4 (PBS) (Fig. 6). Detachment of the biofilm by sodium *m*-periodate supported that these mutant strains, though producing less surface polysaccharide, may contain relatively more EPS in their biofilms. Only the WbtI_G191V_ biofilm was significantly diminished after treatment with DNase (*p* = 0.0190), and WbtI_G191V_Δ1423-22 was the only strain whose biofilm was significantly decreased after treatment with Proteinase K (*p* = 0.0162). These differences indicated that changes in the surface antigen structures on *Francisella* impact the proportional composition of the biofilm matrix, in addition to the amount of biofilm produced.

### Biofilm formation by Type A *F*. *tularensis*

Overall, Type A strains developed less biofilm than LVS or KY99-3387. By 15 days incubation, the Type A strains appeared to have detached and new/continued growth of biofilm was not detected even with the addition of fresh medium, indicating the biofilm lifecycle of Type A strains is shorter than for LVS. The Type A mutant TIGB03 is deficient in O-Ag^25^ and like its LVS counterpart, developed more biofilm compared to parent strain TI0902. After 5 days growth in CDMB, TIGB03 produced significantly more biofilm (~2-fold) than parent strain TI0902 or SCHUS4 in CDMB or in MHB (*p* = 0.0321) (Supplementary Fig. S2). However, by day 10 parent strains SCHUS4 and TI0902 developed prominent biofilms when grown in CDMB that were not significantly different from the biofilm formed by TIGB03 (*p* = 0.78) (Fig. S2). The wildtype strains never developed a prominent biofilm in MHB, but O-Ag mutant TIGB03 did.

### *F. novicida* biofilm

**Figure 7:**
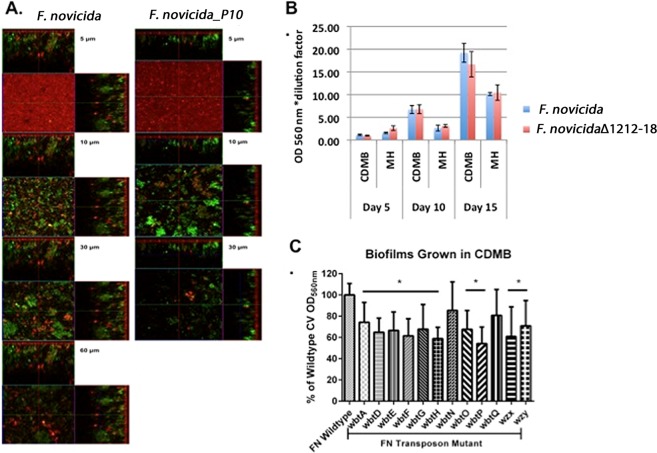
Comparison of biofilm development by *F*. *novicida* and mutant strains. (**A**) CLSM: Orthogonal sections of horizontal (z) and side views (x and y) of reconstructed three-dimensional biofilm images at a magnification of 25x. Nucleic acids were stained with Syto9 (green) and the matrix EPS was stained with Texas Red-Con A (red). Passage of *F*. *novicida* in CDMB to increase CLC production resulted in a less robust biofilm by day 10, but initial attachment did not appear to be affected. Large, dense aggregates of cells were evident by day 10. (**B**) Comparison of biofilm development by *F*. *novicida* and its CLC-deficient mutant grown in CDMB and MHB. No significant differences were seen between parent and mutant biofilms formed in either medium. Like Type B strains, biofilm development peaked at ~15 days. (**C**) Biofilm development of O-Ag mutants of *F*. *novicida*. *F*. *novicida* biofilms were grown for 5 days in CDMB and stained with CV. Data are shown as percent comparison to the parent *F*. *novicida* strain. All O-Ag mutants produced less biofilm than the parent except mutants in the *wbtN* and *wbtQ* genes.

In contrast to the differences in biofilm development observed between Type A strains, the Type B LVS strain, and their surface antigen mutants when grown in CDMB versus MHB, there was not a significant difference in biofilm formation by *F*. *novicida* grown in CDMB or MHB. However, *F*. *novicida* did produce significantly more (*p* < 0.0001) biofilm when the bacteria were grown in BHI or tryptic soy broth (TSB) (OD_560_ 1.5 ± 0.2 and 1.3 ± 0.1, respectively) than in MHB (0.7 ± 0.1). Similar results were obtained for attachment of the cells to microtiter wells (data not shown). As for Type B strains LVS and KY99-3387, increased CLC production (by *F*. *novicida*_P10) negatively affected biofilm formation (Fig. 7A). However, in contrast to parent strain U112, the CLC-deficient mutant *F*. *novicida*Δ1212-1218 did not produce more biofilm than the parent when grown in either medium, but there was a significant increase (~2-fold) in biofilm development when the cells were grown in CDMB compared to MHB (*p* = 0.0050) by days 10 and 15 post-inoculation (Fig. 7B). Surprisingly, most of the *F*. *novicida* O-Ag mutants tested (Supplementary Table S1) made less biofilm than the parent, except for those with mutations in *wbtN* and *wbtQ* (Fig. 7C). *F*. *novicida* also attached more uniformly to the glass coverslip than Type A or LVS strains, including mutants (data not shown). Therefore, there were stark differences in biofilm formation, and the factors that influence biofilm formation, by *F*. *novicida* compared to Types A and B strains, although there were also some similarities.

## Discussion

The capability of *Francisella* species to form biofilms has been confirmed in subspecies *tularensis*, *holarctica*, and *novicida*^12^, but the biofilm made by subspecies *novicida* forms earlier and is denser and more developed than biofilms formed by the other two subspecies. However, details regarding the factors that influence biofilm formation, particularly in Type A and B strains, have not been described. *Francisella* is a unique intracellular pathogen with an extremely low infectious dose, but the role of biofilm formation in the life cycle of this bacterium is unknown. One hypothesis is that biofilm may contribute to survival of *Francisella* spp. outside of the mammalian host^12,26,27^. As a pathogen that can kill most of its hosts, *F*. *tularensis* must survive in a wide range of environmental conditions (hot and arid, cold waterways, and even intracellularly in amoebae)^7,27,28^. The shorter biofilm cycle and overall less biofilm formed by Type A strains, even compared to Type B strains, could be an indicator of the differences in environmental niches that these two biotypes inhabit. Type A strains are found predominately only in drier environments while Type B strains are commonly found in waterways, particularly in Europe, but also in the U.S.^29–31^. The initial attachment and structural stability of biofilms from *F*. *novicida* and Type B strains may need to be thicker and more developed than from Type A strains in order to survive the sheer force of flowing water and the natural predation that occurs in waterways from amoebae and vectors, such as mosquito larvae^2,7,28^. Formation of a biofilm by *Francisella* spp. may also aid survival while inside arthropod vectors^26^. *Yersinia pestis* survives inside the flea gut in biofilm form, and *F*. *novicida* can form biofilms on chitin surfaces^11,32^. Chitin is found in many arthropod vector exoskeletons, and we have found that *F*. *tularensis* Types A/B strains will form biofilms, albeit less prominent (data not shown), at room temperature (~22 °C), which is similar to the internal temperature of a tick^11,33^. Further exploration of this observation is needed.

Besides the subspecies of *Francisella*, the amount of cell surface carbohydrate was found to be the major factor influencing biofilm formation by Types A and B strains. The glycosylated CLC on the cell surface is greatly enhanced when *F*. *tularensis* is serially passed in and grown on medium that simulates *in vivo* growth conditions, such as CDMB^34^. Although LVS mutants that lack CLC on the cell surface are attenuated^20^, biofilm formation by these mutants was also enhanced. When CLC expression was enhanced (by subculture in CDMB) in both LVS and virulent Type B strain KY99-3387, whether O-Ag was present or not, biofilm formation was diminished after 10-15 days incubation. This interference in biofilm formation also occurs in encapsulated bacteria such as *Porphyromonas gingivalis*^35^ and *Pasteurella multocida*^36^. In these species enhanced biofilm formation is associated with loss of capsular polysaccharide. The CLC is composed of multiple glycoproteins, and its expression on the bacterial surface is negatively affected by deletion of two glycosyltransferases in a proposed O-linked protein glycosylation locus (FTT_0789-0800)^20,37^. Similar results have been reported in *Acinetobacter baumannii* lacking PglC, the initiating glycosyltransferases (iGT) in a glycan synthesis locus^38^. The proposed iGT of the *F*. *tularensis* CLC locus, FTT_0790, and the iGT of the O-Ag locus *wbtB*, are homologous to *pglC* in *A*. *baumannii* and could indicate *en bloc* glycan synthesis is involved in CLC O-glycosylation as well^39^.

O-Ag deficient strains of *F*. *tularensis* Types A and B produced 2-5-fold more well-developed biofilms than their more virulent parent strains, yet are attenuated in the highly susceptible mouse model, and compromised in intracellular growth^18,25,40^. *F*. *tularensis* has the ability to phase vary from smooth (blue colonies, O-Ag positive) to rough (grey colonies, O-Ag negative) colony phenotypes^41^, and one potential benefit to this phase variation would be to increase viability in environmental niches through enhanced biofilm formation if O-Ag is lost. Therefore, these results support the hypothesis that phase-variable loss of surface carbohydrates enhance formation of a biofilm, which may contribute to survival of *Francisella* species in the environment, rather than as a virulence mechanism in the host. In other bacterial species truncation of the LPS O-Ag also affects biofilm formation. The loss of O-Ag is thought to result in a change in the cell surface charge, altering cell surface hydrophobicity^42,43^. In *Escherichia coli*, *hld* mutants that express a rough LPS demonstrate an increase in biofilm formation and expression of several surface bacterial factors affecting virulence^44^. Alteration of the cell surface charge has also been shown in *Pseudomonas aeruginosa* and *Bradyrhizobium japonicum* when these bacteria lack O-Ag polysaccharide^45,46^. Differences in biofilm formation by *F*. *novicida* have also been attributed to changes in surface hydrophobicity^26^. However, we were unable to establish any significant differences between the hydrophobicity of *F*. *tularensis* LVS with any of its O-Ag mutants, as all LVS-derived strains and the parent were not incorporated into a hydrophobic hexadecane layer (data not shown). *F*. *tularensis* has a unique O-Ag consisting of two proximal carbohydrate residues (α-D-GalNAcAN–α-D-GalNAcAN) and two distal residues (β-D-Qui4NFm and β-D-QuiNAc), while the *F*. *novicida* O-Ag shares the same two proximal residues, but has different distal glycoses (α-D-GalNAcAN and β-DQui2NAc4NAc)^17^. The difference in O-Ag surface glycoses between the two species could explain, in part, the differences in biofilm formation between the parent strains of *F*. *novicida* and *F*. *tularensis*. While *F*. *novicida* formed a uniform film across the coverslip surface (data not shown), *F*. *tularensis* attached in non-uniform clumps. Removal of the dideoxy O-Ag sugars in *F*. *tularensis* (WbtI_G191V_) allowed for more uniform attachment across the surface, although the biofilm coverage on the coverslip was still not comparable to that of *F*. *novicida*. However, most O-Ag mutants of *F*. *novicida* did not produce more biofilm, as Types A and B O-Ag mutants did, but enhancement or loss of CLC by *F*. *novicida* grown in CDMB did reduce or enhance biofilm formation, respectively, as it did for Type A and B strains.

A double mutant lacking both O-Ag and CLC^20^ formed 7-fold or more biofilm than either the O-Ag mutant or CLC mutant, further supporting an inverse relationship between cell surface-associated glycosylation and biofilm formation. Furthermore, adherence is the first step in biofilm formation, and double mutant WbtI_G191V_Δ1423-22 lacking O-Ag and CLC adhered significantly better to surfaces than cells whose surfaces were glycosylated. Therefore, the presence of glycosylated CLC, O-Ag LPS, and an O-Ag capsule may sterically interfere with protein adhesins^47–49^, thereby reducing adherence and initiation or delay of biofilm formation.

In addition to the O-Ag and CLC, the growth medium also affected biofilm development. Zarrella *et al*.^50^ reported that *F*. *tularensis* undergoes host-adaptation that includes changes in multiple surface polysaccharides, and that these changes can be reproduced by growing *F*. *tularensis* in some, but not all, growth media. Zarrella *et al*. demonstrated that *F*. *tularensis* grown in brain hear infusion broth (BHIB) expresses the same surface polysaccharides when growing in the host, whereas the bacteria did not express these components after growth in MHB. We have determined that *F*. *tularensis* produces the HMS material when grown in CDMB, as well as when it is grown in BHIB, and that this component is associated with the CLC^20,50^. Biofilm formation was also affected when the bacteria were grown in CDMB versus MHB, and an increase in biofilm formation occurred in all surface antigen mutants when grown in CDMB. A significant increase in biofilm formation in WbtI_G191V_Δ1423-22 occurred only when the bacteria were grown in BHIB and CDMB. Overall less biofilm was present when the bacteria were grown in MHB, and the differences observed in the CLC-deficient mutant grown in BHIB were not found in the double mutant WbtI_G191V_Δ1423-22 grown in MHB. Therefore, there are likely factor(s) available in BHIB or CDMB that stimulate biofilm formation, possibly by upregulating adherence factors. Of interest was that growth of *F*. *novicida* in CDMB did not enhance biofilm formation, but growth in BHIB and TSB did. These differences on factors that influence biofilm formation between *F*. *novicida* and Types A and B strains may be related to genetic regulators that are governed by environmental adaptations required by the different species.

Mutations in the only two polysaccharide loci thus far identified in *Francisella* positively enhanced biofilm production, questioning what the composition is of the EPS forming the biofilm matrix. The *F*. *tularensis* biofilm is susceptible to enzymes that degrade DNA, proteins, and/or polysaccharide, and therefore is likely to contain each of these components at some stage in development. Cywes-Bentley *et al*. have reported that a monoclonal antibody to β-(1,6)-linked poly-*N*-acetyl-D-glucosamine (PNAG), which is a common EPS in the biofilms of many bacterial species, reacts with *F*. *tularensis*^51^. However, our attempts to detect PNAG in the *F*. *tularensis* biofilm using a fluorescent-tagged wheat germ agglutinin lectin (WGA) (specific to PNAG), or antibodies to PNAG were unsuccessful. Extracts from *F*. *tularensis* biofilm as well as CLC were also incubated with fluorescein-tagged WGA, but no fluorescence was detected (data not shown). Phenol extraction of the EPS from the most prominent biofilm-forming strain, WbtI_G191V_Δ1423-22, and subsequent compositional and linkage analysis indicated the EPS consisted predominately of end-linked and 1,4-linked glucose, suggesting that the EPS was similar to amylose. Glycogen expression and regulation in *E*. *coli* is controlled by the genes within the operon *glgBXCAP*^52^. Proteins with low identity or similarity to GLGBXCAP were identified in *F*. *tularensis*. In addition, *F*. *tularensis* gene FTT_1629c, which annotated as a hypothetical gene, had 25.8% identity to the *Streptococcus pneumoniae tts* gene, which is solely responsible for producing the type 37 capsule that is comprised of (1,3)-Β-glucan^53^. FTT_1629c also includes a DXD motif (glycose binding domain), which indicates it is likely a glycosyltransferase. Alternatively, there may be more than one *F*. *tularensis* EPS, and the predominant EPS of the WbtI_G191V_Δ1423-22 mutant may differ from that of Type A strains. One of the glycosyl transferases deleted in WbtI_G191V_Δ1423-22, FTL_1423, has some identity to the *Y*. *pestis hmr* gene, which is similar to *icaA*, an *N*-glycosyltransferase used for PNAG production^54^.

In conclusion, lack of the surface antigens CLC, LPS O-Ag, and O-Ag capsule resulted in poorer biofilm formation by *F*. *tularensis* Types A and B *in vitro*. Strains lacking one or all surface carbohydrate antigens formed a more robust biofilm than the parent strain, and such loss could occur naturally. Biofilm formation was also affected by incubation time and the specific growth medium. For the first time, the extended biofilm development cycle of *F*. *tularensis* Types A and B strains has been described, a previously unreported glucan polysaccharide that makes up the biofilm matrix has been identified, and cell-associated surface polysaccharides of *F*. *tularensis* Types A and B were shown to interfere with biofilm development, which is not required for bacterial virulence.

## Methods

### Bacterial strains and growth conditions

The strains used in this study are described in Table 1. Generation of mutants WbtI_G191V_ and LVSΔ1423-22 and their complementation to obtain WbtI_G191V_^+^ and LVSΔ1423-22^+^ has been previously reported^18,20^. Strains were cultured on either CDMB with 1.5% glucose, BHIB, TSB, or MHB (BD, Franklin Lakes, NJ) (all supplemented with 0.1% cysteine) at 37 °C in 6% CO_2_^34^. To enhance CLC expression the bacteria were subcultured in CDMB for 10 subsequent passages, then grown on CDMB agar at 32 °C in 6% CO_2_ for 5 days, as previously described^20^. All subcultured strains, parents, and mutants were stored at −80 °C in sterile 10% skim milk until needed and were not subcultured unintentionally. Recombinant strains were grown in CDMB with 15 μg/ml kanamycin (Kan). All experiments with LVS, *F*. *novicida*, and mutants were carried out in biosafety level (BSL)-2 facilities in an approved biosafety cabinet. All Type A strains were grown in BSL-3 facilities, and removed from the facility only when loss of viability was confirmed by subculture and 5 days incubation.

### Static biofilm formation

A modified version of the method by Stepanovic *et al*.^55^ was used to grow biofilms in round-bottomed, poly-L-lysine-coated, polystyrene microtiter plates (Nunc, Rochester, NY). Briefly, overnight cultures of *F*. *tularensis* strains were grown at 37 °C and stationary phase bacteria were diluted 1:4 in either CDMB or MHB. The samples were incubated for 5, 10, 15, 20, or 25 days at 37 °C, and the medium replaced every 5 days. The attached biofilms were quantified by solubilising the CV with 95% ethanol and determining the absorbance at 560 nm. For plates with sample absorbance values greater than 4.0, a 1:3-1:10 dilution was made of all samples on the plate. All results involving the CV biofilm assay with mutant strains were calculated and compared to the parent strain and blank control in the same 96-well plate to eliminate technical bias.

### Attachment assay

Triplicate cultures of all strains were grown with shaking (180 rpm) overnight at 37 °C to stationary phase. Cultures were transferred (200 μl) to 96-well polystyrene plates that had been coated with poly-L-lysine (Lab-Tek II/Thermo Fisher Scientific, Waltham, MA) and allowed to adhere for 1, 2, or 4 hours statically at 37 °C. The medium was removed and the wells stained with CV as described above. All assays were repeated in triplicate.

### Confocal laser scanning microscopy and image analysis

Biofilms were statically grown for 5, 10, or 15 days on 8-well chambered glass coverslips coated with poly-L-lysine (Lab-Tek II/Thermo Fisher Scientific). Medium and non-adherent cells were replaced with fresh medium every 5 days (flow cells were not used due to restrictions in the BSL-3 laboratory). At each time point, the medium was removed from the well, the coverslip was washed with sterile saline twice, and 40 μM/ml Syto9 in sterile saline from the BacLight stain (Molecular Probes, Inc., Eugene, OR) or 50 μg/ml Texas Red-labeled ConA in saline was added. After 15 min incubation at room temperature in the dark, the coverslip wells were washed in saline and visualised by CLSM using a ZEISS LSM 510 laser-scanning microscope, mounted on an inverted Axio Observer Z1 (Carl Zeiss, Jena, Germany). Laser scanning image browser software was used for analysis of the biofilm images, including collection of z-stacks, three-dimensional reconstruction, and data analysis. Images were acquired from the center of the coverslip in 1.1-μm sections throughout the biofilm depth, with the number of sections varying depending on the thickness of the biofilm.

### Electron microscopy

Each strain was grown as a biofilm on 10-mm diameter silicone disks in CDMB for 5 days under static conditions at 32 °C for examination by scanning electron microscopy (SEM). After removal of the medium, biofilms were fixed in 2% glutaraldehyde, followed by 1% osmium tetraoxide for 30 minutes each. The samples were dehydrated in a sequential series of ethanol solutions (increasing from 10% to 100%). The disks were critical point-dried, sputter-coated with carbon, and examined using a Zeiss EVO40 scanning electron microscope.

### Exopolysaccharide isolation and analysis

EPS was isolated from LVS mutant WbtI_G191V_Δ1423-22, which lacked both O-Ag and CLC glycosylation, and formed the most robust biofilm. The strain was grown statically in 200-ml volumes of BHIB or CDMB supplemented with 1% glucose, in large tissue culture flasks coated with poly-L-lysine at 32 °C or 37 °C for 10-15 days. The supernatant was carefully removed, the sediment extracted with 45% aqueous phenol, and the EPS purified as previously described^56^.

Glycose composition analysis was performed by GC-MS of the per-*O*-trimethylsilyl (TMS) derivatives of the monosaccharide methyl glycosides produced from the sample by acidic methanolysis, as described^57^. Briefly, 300 μg of sample was added to a tube containing 20 µg of inositol as an internal standard. Methyl glycosides were prepared from the dry sample following methanolysis in 1 M HCl in methanol at 80 °C (16 hours), followed by re-*N-*acetylation with pyridine and acetic anhydride in methanol (for detection of amino sugars). The sample was then per-*O*-trimethylsilylated by treatment with Tri-Sil (Pierce) at 80 °C for 30 min. Gas chromatography-mass spectrometry (GC/MS) analysis of the TMS methyl glycosides was performed on an Agilent 7890 A GC interfaced to a 5975 C mass selective detector using Agilent DB-1 fused silica capillary column (30 m × 0.25 mm ID). For glycose linkage analysis, approximately 1 mg of the sample was suspended in 200 μl of dimethyl sulfoxide, and stirred for 1 day. The sample was permethylated as described by Ciucanu and Kerek^58^ with the base prepared according to Anumula and Taylor^59^. Briefly, the sample was subjected to the NaOH base for 15 min, followed by addition of methyl iodide and left for 45 minutes. This procedure was repeated one more time to ensure complete methylation of the polymer. The permethylated material was hydrolysed using 2 M trifluoroacetic acid (2 h in sealed tube at 121 °C), reduced with NaBD_4_, and acetylated using acetic anhydride/TFA. The resulting partially methylated alditol acetates were analysed on the same equipment described above, except separation was performed on a 30 m Supelco SP-2331 bonded phase fused silica capillary column, as described by York *et al*.^60^. Identification of genes that may be involved in expression and regulation of glycogen synthesis was determined by BLASTP^61^.

### Enzymatic detachment assays

Biofilms were grown in CDMB for 10 days in 96-well plates as described above. Wells were washed once with PBS and the biofilm treated with Proteinase K (100 μg/ml in 10 mM Tris-HCl pH 7.5, Sigma-Aldrich), 40 mM sodium *m*-periodate, or DNase I (100 μg/ml in 150 mM NaCl/1 mM CaCl_2_) (Sigma-Aldrich). Enzymes were added to designated wells in a volume of 200 μl and incubated at room temperature for 2 hours. Enzyme solutions were removed and the wells washed once with PBS and then stained with CV, as described above. All assays were performed in triplicate unless otherwise stated.

### Analysis of large molecular size components associated with CLC

*F*. *tularensis* strains were grown with shaking at 37 °C overnight in either CDMB or MHB and 500 μl was used to inoculate 10 ml of new MHB or CDMB in 50-ml conical tubes (Corning, Oneonta, NY). The tubes were held stationary at 37 °C for 10 days to allow bacteria to adhere and form biofilms. The medium above the biofilm was then removed (broth supernatant) and non-adherent, planktonic cells were sedimented by centrifugation at 6,000 × *g* for 15 min. Proteins in the supernatant were precipitated by addition of 3 volumes of 95% ethanol, sedimented by centrifugation (as above), resuspended in a small volume of distilled water, and the protein concentration determined by BCA assay (Thermo Fisher Scientific). Surface components of the biofilm and planktonic cells were extracted by suspending each bacterial phenotype in 1–5 ml of 1 M urea and holding the bacteria without shaking at room temperature for 15 min. The cells were removed by centrifugation (6,000 × *g* for 15 minutes) and the urea extracts stored at −20 °C until analysed. Solubilisation buffer (2×) was added to an equal volume of the urea extract and 15 μl was loaded onto 4–12% NuPAGE gels (Invitrogen/Thermo Fisher Scientific). Following electrophoresis the gels were stained for proteins with silver stain (Pierce, Rockford, IL), for carbohydrates with Pro-Q Emerald 300 (Molecular Probes, Grand Island, NY), or for CLC with StainsAll/silver stain (Pierce/Sigma-Aldrich)^20^.

### Statistical analyses

Statistical analyses were performed using Microsoft Excel software (Redmond, WA) and Prism GraphPad 6 software (San Diego, CA). Data obtained with LVS strains were analysed using the student t-test and expressed as the mean ± standard deviation. Results with a *p* value of less than 0.05 were considered statistically significant. One-way ANOVA was used to evaluate significant differences in CV absorbance of *F*. *novicida* and the *F*. *novicida* O-Ag mutants. Dunnet’s multiple comparison test was performed after completion of the one-way ANOVA to determine significance between the CV absorbance of *F*. *novicida* with the absorbance of a specific *F*. *novicida* O-Ag mutant. Adjusted *p*-values were determined for each comparison.

## Supplementary information

Supporting Information Revised

## Data Availability

The datasets generated during and/or analysed during the current study are available in the U.S. National Library of Medicine National Center for Biotechnology Information repository (https://blast.ncbi.nlm.nih.gov/Blast.cgi?PAGE=Proteins). Other data generated or analysed during this study are included in this published article (and its Supplementary Information files), or available from the corresponding author on reasonable request.
