# Supplementary material for: Formation of the Francisella tularensis Biofilm is Affected by Cell Surface Glycosylation, Growth Medium, and a Glucan Exopolysaccharide
Source: Sci Rep. 2019 Aug 22;9:12252. doi: 10.1038/s41598-019-48697-x (PMC6706388; doi:10.1038/s41598-019-48697-x)
Supplement: Supplementary file 1 — Supporting Information Revised [file 41598_2019_48697_MOESM1_ESM.pdf]

Formation of the *Francisella tularensis* Biofilm is Affected by Cell Surface Glycosylation, Growth Medium, and a Glucan Exopolysaccharide

Anna E. Champion, Kelly C. Freudenberger Catanzaro, Aloka B. Bandara, and Thomas J. Inzana

Supporting Information

Supplementary Table S1. ***Francisella novicida*** **transposon library mutant strains used.**

**Bacterial Strain**	**Locus Tag**	**Gene Name**	**Description**
tnfn1_pw060328p01q189	FTN_1420	*wzx*	O antigen flippase
tnfn1_pw060323p06q119	FTN_1421	*wbtH*	Glutamine amidotransferase/asparagine synthase
tnfn1_pw060323p06q136	FTN_1422	*wbtN*	Glycosyl transferase, group 1
tnfn1_pw060323p03q189	FTN_1423	*wbtG*	Glycosyl transferase, group 1
tnfn1_pw060328p05q111	FTN_1424	*wzy*	Hypothetical membrane protein
tnfn1_pw060323p06q161	FTN_1425	*wbtF*	NAD-dependent epimerase
tnfn1_pw060328p03q164	FTN_1426	*wbtE*	USD-glucose/GDP-mannose dehydrogenase family protein
tnfn1_pw060419p04q192	FTN_1427	*wbtD*	Glycosyl transferase, group 1
tnfn1_pw060323p03q182	FTN_1428	*wbtO*	Transferase
tnfn1_pw060328p05q149	FTN_1429	*wbyP*	Galactosyl transferase
tnfn1_pw060419p04q158	FTN_1430	*wbtQ*	Aminotransferase
tnfn1_pw060323p06q123	FTN_1431	*wbtA*	dTDP-glucose 4,6-dehydratase

Supplementary Fig. S1. Glycose composition and linkage analysis of the EPS from *F. tularensis* by gas-chromatography-mass spectrometry (GC-MS). The purified EPS was permethylated, hydrolyzed, reduced, and acetylated as described in Methods. The methylated alditol acetates were separated on a 30 m Supelco SP-2331 bonded phase fused silica capillary column, and analyzed on an Agilent 7890A GC interfaced to a 5975C mass selective detector. Glycose analysis by GC-MS (top panel) showed that 2 monosaccharides were identified, consisting of 99.9% glucose and 0.1% mannose. Linkage analysis (bottom panel) indicated that 54% of the glucose residues were terminal, 33% were 1,4-linked, and 1.6% were 1,6-linked, which is consistent with amylose. A minor component (4.4%) of the EPS was 1,4-linked mannose.

Supplementary Fig. S2. Static biofilm development by *F. tularensis* Type A strains in A: MHB or B: CDMB. Differences in biofilm development between Type A strains SCHUS4 and clinical isolate TI0902 were not significant whether grown in MH medium (A) or CDM medium (B) ($p > 0.5$). However, O-Ag mutant TIGB03 made significantly more biofilm after 5 and 10 days incubation in MH medium ($p < 0.01$), and after 5 days incubation in CDM medium (surface $p < 0.05$). However, the amount of biofilm made by SCHUS4 and TI0902 increased enough by day 10 that there was not a significant difference in biofilm formation. Significance was determined by unpaired *t* test.

Supplementary Fig. S3. Surface and broth extractions of LVS strains grown as biofilms. Each bacterial strain/mutant was grown in CDMB for 25 days, the medium above the biofilm was removed, the planktonic cells harvested by centrifugation, proteins in the medium precipitated by addition of 3 volumes of 95% ethanol, and the precipitate resuspended in distilled water. Planktonic cells in the medium and cells in the biofilm were extracted with 1M urea. The protein profiles were resolved by SDS-PAGE on 4-12% gels, stained with silver. L: LVS, P: LVS_P10, LM: LVSΔ1423-22, W: WbtIG191V, WM: WbtIG191VΔ1423-22, M: Ladder.

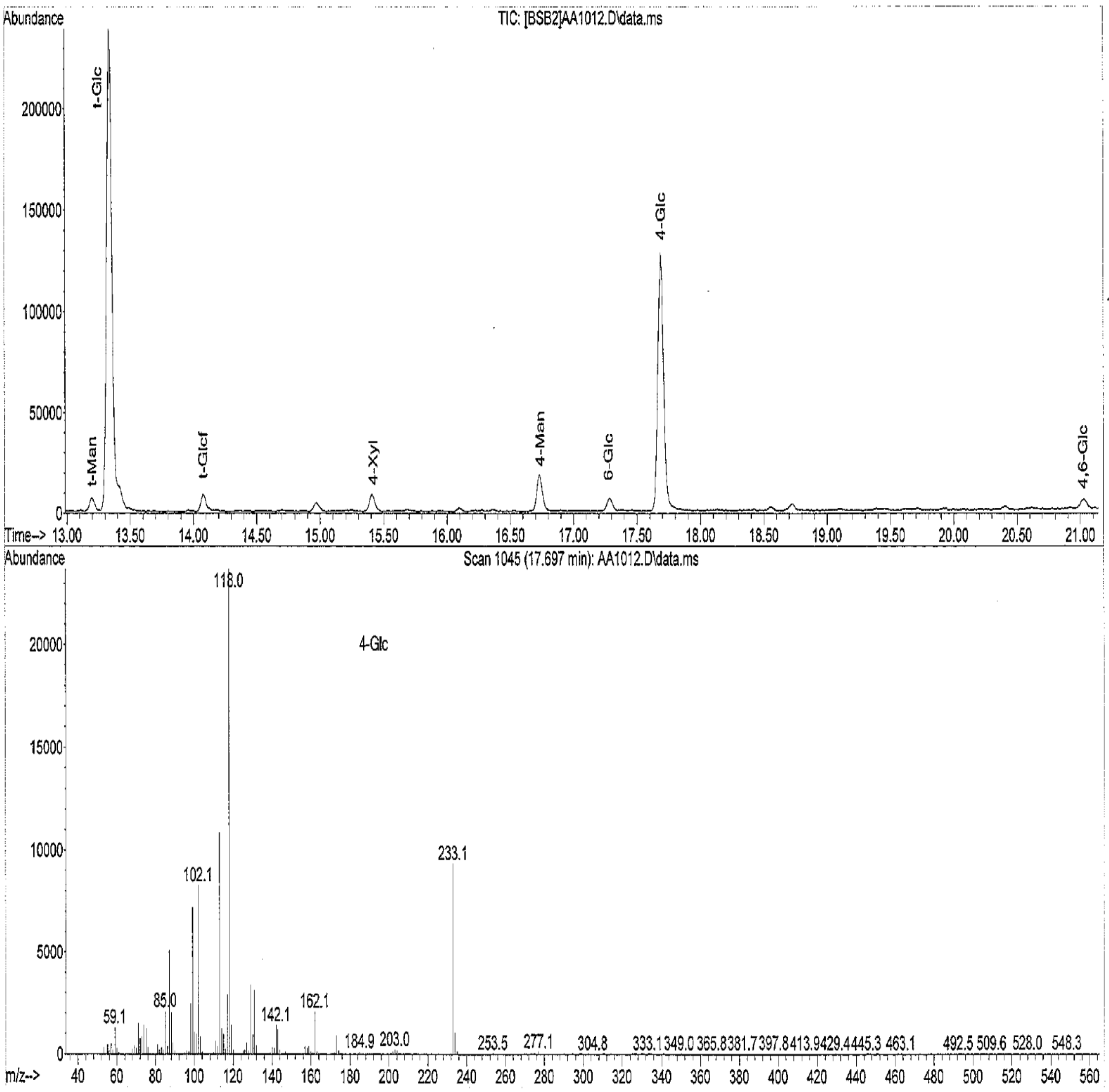

Supplementary Fig. S1.

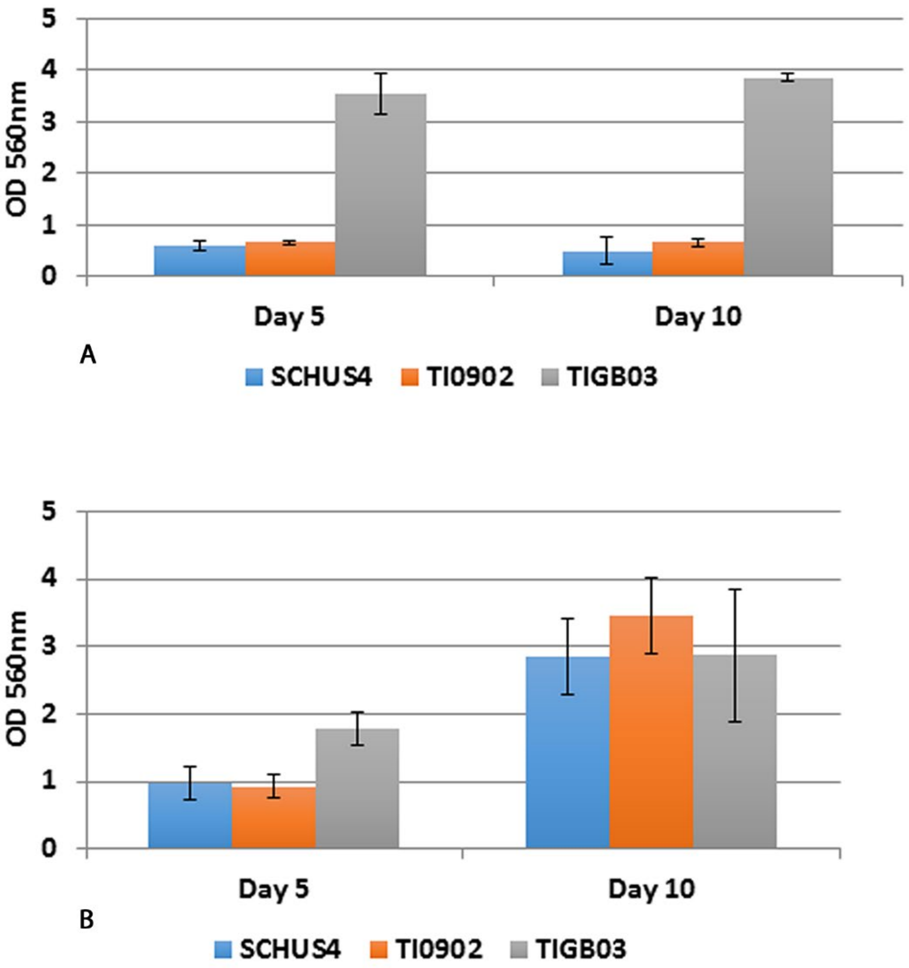

Supplementary Fig. S2.

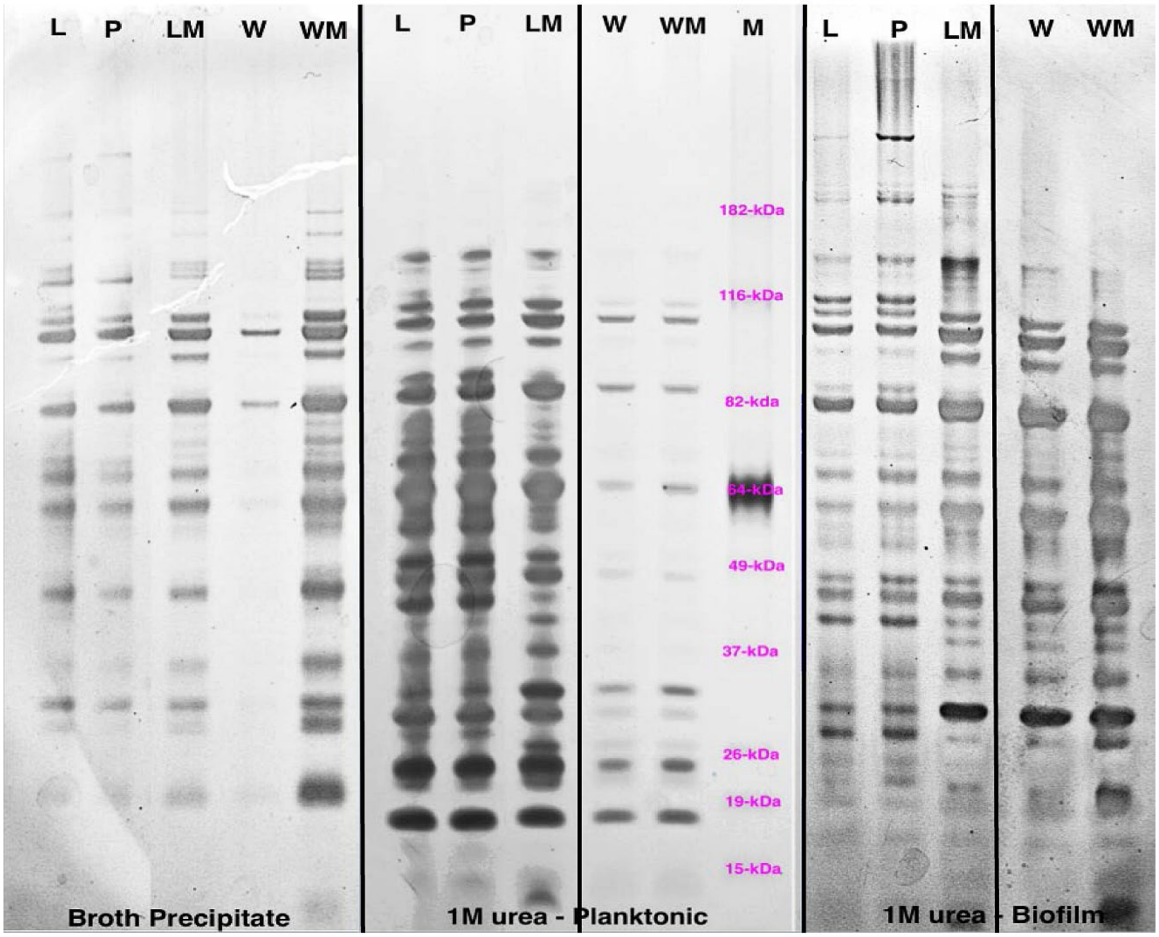

Supplementary Fig. S3.
